# Insights from the first genome assembly of Onion (*Allium cepa*)

**DOI:** 10.1093/g3journal/jkab243

**Published:** 2021-07-13

**Authors:** Richard Finkers, Martijn van Kaauwen, Kai Ament, Karin Burger-Meijer, Raymond Egging, Henk Huits, Linda Kodde, Laurens Kroon, Masayoshi Shigyo, Shusei Sato, Ben Vosman, Wilbert van Workum, Olga Scholten

**Affiliations:** 1 Plant Breeding, Wageningen University and Research Centre, 6700 AA Wageningen, The Netherlands; 2 Bejo Zaden B.V., 1749 CZ Warmerhuizen, The Netherlands; 3 GenomeScan, 2333 BZ Leiden, The Netherlands; 4 Laboratory of Vegetable Crop Science, College of Agriculture, Graduate School of Sciences and Technology for Innovation, Yamaguchi University Yamaguchi City, Yamaguchi 753-8515, Japan; 5 Graduate School of Life Sciences, Tohoku University, Sendai 980-8577, Japan; 6 Limes Innovations B.V., 2351 SX Leiderdorp, The Netherlands

**Keywords:** large genome, repeats, gene space, DHCU066619

## Abstract

Onion is an important vegetable crop with an estimated genome size of 16 Gb. We describe the *de novo* assembly and *ab initio* annotation of the genome of a doubled haploid onion line DHCU066619, which resulted in a final assembly of 14.9 Gb with an N50 of 464 Kb. Of this, 2.4 Gb was ordered into eight pseudomolecules using four genetic linkage maps. The remainder of the genome is available in 89.6 K scaffolds. Only 72.4% of the genome could be identified as repetitive sequences and consist, to a large extent, of (retro) transposons. In addition, an estimated 20% of the putative (retro) transposons had accumulated a large number of mutations, hampering their identification, but facilitating their assembly. These elements are probably already quite old. The *ab initio* gene prediction indicated 540,925 putative gene models, which is far more than expected, possibly due to the presence of pseudogenes. Of these models, 47,066 showed RNASeq support. No gene rich regions were found, genes are uniformly distributed over the genome. Analysis of synteny with *Allium sativum* (garlic) showed collinearity but also major rearrangements between both species. This assembly is the first high-quality genome sequence available for the study of onion and will be a valuable resource for further research.

## Introduction

More than just a tasty culinary sensation, onion (*Allium cepa* L.) is one of the most important vegetable crops worldwide. In terms of global production value, onion ranks second after tomato (http://www.fao.org/faostat/en/#home). Onion is a diploid (2*n* = 2*x* = 16) species with a genome size of approx. 16,400 Mb/1C (Van’T Hof 1965; Arumuganathan and Earle 1991; Ricroch *et al.* 2005), the largest of all cultivated diploid crops and of a size comparable to the allo-hexaploid bread wheat (Brenchley *et al.* 2012; Marcussen *et al.* 2014). A large genome size is often associated with repeat accumulation (Kelly and Leitch 2011). The C_o_T reannealing kinetics indicate that about 40% of the onion genome is highly repetitive (>1000× copies) and 40% has 100–1000 copies and is thus middle to low repetitive (Stack and Comings 1979). Overall, at least 95% of the *A. cepa* genome consists of repetitive sequences (Flavell *et al.* 1974), most of which are dispersed repeats (Shibata and Hizume 2002) and LTR retrotransposons of the Ty1/copia and Ty3/gypsy type (Pearce *et al.* 1996; Kumar *et al.* 1997; Pich and Schubert 1998; Vitte *et al.* 2013). Due to the size of the genome and the repetitive nature, developing an onion reference genome assembly is challenging (Havey and McCallum 2012).

For onion, molecular breeding strategies are currently limited to the use of molecular markers and genetic linkage maps (Martin *et al.* 2005; McCallum *et al.* 2012; Baldwin *et al.* 2012; Scholten *et al.* 2016). Knowledge of the genome of onion and related species is scarce compared to other crop plants, with only transcriptome sequences available (Kim *et al.* 2014; Kamenetsky *et al.* 2015; Sohn *et al.* 2016; Abdelrahman *et al.* 2017). While the availability of a reference genome has greatly stimulated research and led to accelerated breeding in many other crops (Kuhl *et al.* 2005; Vitte *et al.* 2013; Sun *et al.* 2020), onion has not yet had this benefit. Garlic (*Allium sativum*; 16.2 Gb) and asparagus (*Asparagus officinalis* L.; 1.1 Gb) are the most closely related species with reference genomes available (Harkess *et al.* 2017; Sun *et al.* 2020). Though, useful for gene discovery, the lack of insight into detailed syntenic relationship between these crops and onion is still limiting the utilization in onion (breeding) research.

In this article, we describe the first *de novo* assembly of the genome of a doubled haploid *A. cepa* accession through a combination of strategies and the development of an initial set of pseudomolecules. Synteny between onion and garlic was studied and provides a first insight into the similarities and differences in genome organization. This onion assembly will be an important tool, facilitating onion breeding and research.

## Materials and methods

### Plant material

Seeds of the doubled haploid (DH) *Allium cepa* line DHCU066619 (Hyde *et al.* 2012) were kindly provided by Dr. M. Mutschler (Cornell University). This DH accession was selected for whole-genome shotgun sequencing (WGS), as it is a homozygous, vigorously growing genotype.

### DNA and RNA isolation

Two grams of leaf tips from young and rapid-growing onion plants were harvested and pooled for nuclei DNA isolation according to the protocol described by Bernatzky and Tanksley, (1986).

For RNA extraction, tissues from bulb and basal plate, as well as leaf and flowers from mature plants were harvested, frozen in liquid nitrogen and grinded. Approximately 100 mg of each tissue was transferred to a 2 ml screwcap tube, followed by adding 800 µl of Trizol and vortexing for 1 min. Subsequently, 160 µl of Chloroform was added, and gently mixed for 15 s. After incubating the samples for 3 min at RT, they were centrifuged at 10,000 rpm in an Eppendorf centrifuge for 3 min, followed by transfer of the water-phase to a clean tube. A 350 µl of RLT buffer (Qiagen) containing 10 µg/µl β-mercaptoethanol, was added to each 100 µl of water-phase, followed by a RNeasy (Qiagen) column extraction.

### Sequencing methods, preparation details, and data processing

Three TruSeq sequencing libraries (with median insert sizes of 230, 350, and 500) were made according to the manufacturer’s recommendations and sequenced during a single Illumina^®^ HiSeq 2500 run (across 16 lanes) by GenomeScan (Leiden, The Netherlands). Quality of the data was assessed using fastQC (https://www.bioinformatics.babraham.ac.uk/projects/fastqc/) and by evaluating the k-mer profile using JELLYFISH (Marçais and Kingsford 2011).

PacBio Sample preparation was performed according to the PacBio protocol “*20 kb Template Preparation Using BluePippin™ Size-Selection System*.” In short, 8 µg of sample was fragmented using a Covaris g-Tube at 4800 rpm for 1 min. The PacBio SMRTbell™ Template Prep Kit 1.0 was used for the DNA library preparation of the primer annealed SMRTbells. The SMRTbells were size selected using the BluePippin set for 10 kb–50 kb long reads. The PacBio DNA/Polymerase Binding Kit P6 was used to bind prepared SMRTbell libraries to the DNA polymerase in preparation for sequencing on the PacBio *RS* II. The complex of polymerase bound SMRTbell was mixed with long-term storage buffer. Two batches of stored complex were made. Prior to sequencing the SMRTbell complex was incubated with PacBio MagBeads. Sequencing was performed with the PacBio DNA Sequencing Kit 4.0 chemistry. For the sequencing run MagBead loading and Stage Start were enabled. Twenty sequencing runs, totaling 138 SMRTcells, were performed with a 360-min movie time per SMRTcell. The sequence runs were performed on the PacBio RS II sequencer and primary analysis was performed with the SMRT Analysis server version 2.3.0 (GenomeScan, The Netherlands).

### Genome assembly

Illumina reads were assembled using the MaSuRCA 2.3.1 assembly pipeline according to the author’s recommendations (Zimin *et al.* 2013). The PacBio reads were assembled with the Illumina assembly as a backbone using DBG2OLC (Ye *et al.* 2016) and varying settings for kmerSize, KmerCov, MinOverlap, and AdaptTH (Supplementary Files S2 and S3). Final DBG2OLC assembly was performed with KmerSize = 21, KmerCov =2, MinOverlap = 20, and AdaptThr = 0.05.

### Genome scaffolding

#### Dovetail scaffolding

Five grams of young leaf tissue (lyophilized) was sent to Dovetail Genomics (USA) for High Molecular Weight (HMW) DNA isolation, together with the PacBio/Illumina hybrid assembly, the unplaced Illumina scaffolds >3 Kb and a published onion chloroplast sequence (von Kohn *et al.* 2013) to produce a scaffolded assembly according to the Chicago protocol (Putnam *et al.* 2016). For this, the generated DOVETAIL sequencing libraries were analyzed with a modified version of the HiRise algorithm (Dovetail Genomics, USA) to accommodate the large genome size.

#### Genetic marker-based scaffolding

The dovetail scaffolded genome assembly was anchored into pseudomolecules with Allmaps (Tang *et al.* 2015) using four previously published genetic linkage maps (Duangjit *et al.* 2013; Scholten *et al.* 2016; Choi *et al.* 2020; Fujito *et al.* 2021). For the DHAxDHC genetic map (Fujito *et al.* 2021), mean genetic position of the bin was used for all markers in this bin.

### Genome annotation

#### Completeness

Completeness of the onion genome assembly was assessed by BUSCO v4.1.1; database version embryophyte_obd10 2019-11-27 (Simao *et al.* 2015).

#### Repeat annotation

To structurally annotate repeat sequences in the *A. cepa* genome *de novo*, RepeatMasker was applied using the REPBASE v20.5 library. *Ab initio* prediction of repeats was performed using the TEdenovo pipeline of REPET v2.5 (Quesneville *et al.* 2005; Flutre *et al.* 2011), with default parameters, utilizing NCBI-Blast+. To reduce computational time needed to execute the TEdenovo pipeline, 0.52 Gb of the 14.4 Gb (3.6%) of the onion assembly was selected at random. Grouper, Recon, and Piler steps were invoked both with and without structural detection. Repbase v20.05 and Pfam27.0 HMM profiles were used to annotate repeats identified in onion by the pipeline. The output of the TEdenovo pipeline was subsequently used as the reference library to run the TEannot pipeline on randomized chucks of the whole assembly using default parameters and exported to GFF3 format.

### *Ab initio* gene prediction

RNA from four tissues (bulb, basal plate, leaf, and flower) was isolated and sequenced by GenomeScan (Leiden, The Netherlands) using an Illumina HiSeq 2500 sequencer according to the manufacture’s recommendations. BRAKER1 (Hoff *et al.* 2015) was used for unsupervised training of Augustus 3.2.2 (Stanke *et al.* 2008), using splice junctions identified from mapping RNAseq reads with STAR mapper (Dobin *et al.* 2013) of all four tissues combined. Subsequently, the training parameters from BRAKER1 were used to annotate the masked genome sequence.

### Functional annotation/Blast2go interpro

Blast2Go (Gotz *et al.* 2008) was used for functional annotation of the predicted protein models with the default settings for the mapping and annotation step. The initial blastp 2.6.0+ step was performed against the Swissport database (version 4 October 2017) with an e-value cutoff of 1.0E^−3^, word size of 6, Low Complexity filter on true, and a maximum of 20 blast hits. InterProScan v5.26 (Jones *et al.* 2014) including panther 12.0 libraries was used to identify protein domains within the predicted protein sets.

### Synteny analysis

Sequence of EST-based markers from Fujito *et al.* (2021) were blasted to the onion genome and to the garlic genome. A total of 4034 markers had a hit to both genome sequences, of which 519 positioned at unanchored onion scaffolds, leaving 3515 markers positioned at the onion pseudomolecules. Based on the top hits, the physical positions of markers with a match to both genomes were plotted against each other in a XY plot using the python matplotlib and seaborn libraries.

## Results and discussion

### Genome assembly

The doubled haploid *Allium cepa* accession DHCU066619 (Hyde *et al.* 2012) was selected for WGS, to facilitate genome assembly especially of a large genome like onion. Three small insert libraries were used for Illumina HiSeq 2500 sequencing (Supplementary Table S1) and yielded 769 Gb sequence data. Analysis of the sequence libraries resulted in ∼450 G *k*-mers (*k* = 31), and an estimated genome size of approx. 13.6 Gb. Of this, approx. 7.4 Gb (53.8%) is single copy based on *k*-mer statistics, indicating that an initial assembly from Illumina reads is feasible. The MaSurCa based assembly resulted in 10.8 Gb in 6.2 M contigs with a contig N50 of 2.7Kb (Table 1). This assembly was further scaffolded using 18.1 M PacBio RS II reads >1 KB with DBG2OLC. This resulted in an assembly of 14.6 Gb in 316 K contigs and a contig N50 size of 59 Kb (Table 1). In the last step, the hybrid Illumina/PacBio assembly was further improved using Dovetail Chicago and subsequent HiRise scaffolding which indicated that there were no mis-assemblies in the Illumina/PacBio hybrid contigs, showing the high quality of this initial assembly. The combination of these three technologies resulted in an assembly of 14.9 Gb in 92.9 K scaffolds with a scaffold N50 size of 454 Kb. With an estimated genome size of 16,400 Gb/1C, we managed to assemble ∼91% of the onion genome. As our assembly is mostly based on short read Illumina sequencing, with limited data from third generation PacBio long reads, we hypothesize that the most complex highly repetitive regions are missing from our assembly.

**Table 1:** Statistics of the steps in the onion genome assembly process

	Illumina	Illumina and PacBio hybrid	Illumina, PacBio, and Dovetail	Illumina, PacBio, Dovetail, and Allmaps—*A. cepa* v1.2
Total assembly size (GB)	10.80	14.59	14.94	14.94
N50 (bp)	2776	59,009	436,203	464,272
L50	91,028,083	76,344	9266	6486
NR of contigs (K)	6200	316	—	—
NR of scaffolds (K)	—	—	92.9	89.6
NR of scaffolds incorporated in pseudomolecules (K)	—	—	—	3.3
Assembly size in pseudomolecules (Gb)	—	—	—	2.4
G + C content (%)	33.2	33.5	33.5	33.5
Estimated TE content (%)	—	—	—	72.4
Number of gene models	—	—	—	540,925
Number of gene models with >90% RNAseq support	—	—	—	47,066
Median/average/max exon length (bp)*a*	—	—	—	137/202/14,836
Median/average/max intron length (bp)*a*	—	—	—	178/1035/213,043
*N*’S per 100 Kbp	0	0	2344	2346

a Gene statistics based on 86k models with blast support.

### Anchoring scaffolds into pseudomolecules using multiple EST-based genetic maps

To further organize our genome assembly, we used three intraspecific genetic linkage maps (Duangjit *et al.* 2013; Choi *et al.* 2020; Fujito *et al.* 2021) and one interspecific genetic linkage map (Scholten *et al.* 2016) to anchor scaffolds into pseudomolecules using AllMaps (Tang *et al.* 2015). Except for the GBS-based markers from the study of Choi *et al.* (2020), all markers were developed from transcriptome sequencing. With this approach, we were able to anchor 3303 scaffolds (3.6% of the total number of scaffolds) into eight pseudomolecules, with an overall length of 2.4 Gb (15.9% of the assembly size; Supplementary File S6). The pseudomolecules were named after their respective chromosome according to the *A. cepa* monosomic addition lines (Shigyo *et al.* 1996; Van Heusden *et al.* 2000; Fujito *et al.* 2021). A subset of 157 scaffolds (0.15 Gb) could be oriented according to the genetic map order. Overall agreement between the scaffolds and genetic positions was good (Supplementary File S6) with absolute Spearman correlation coefficients over the four maps ranging between 0.86 (BYGxAC) and 0.95 (DHAxDHC). While the overall order of chromosome 8 over all four maps was very consistent (Spearman correlation coefficient of 0.99), chromosome 7 showed the lowest correlation (Spearman rho coefficient of 0.716), mainly because of the interspecific CCxRF map (ρ = 0.428). Disruption of collinearity in homoeologous chromosomes in *Allium* species has been previously reported (Khrustaleva *et al.* 2019a). Not all maps were informative in the Allmaps scaffolding. Markers of the CCxRF map did not contribute to the scaffolding of chromosome 3 while markers of the GBS map did not contribute to the scaffolding of chromosome 3, 5, and 8. ALLMAPS has implemented an algorithm to minimize ambiguity (Tang *et al.* 2015), and the GBS marker-based sequences showed similarity to more than one scaffold, which were assigned to different linkage groups, and were therefore skipped for the final scaffolding. This resulted in v1.2 of the genome assembly. Overall, the developed pseudomolecules will be very useful in developing additional genetic markers, fine mapping QTL regions and/or candidate gene mining.

### Genome annotation

The completeness of the Dovetail scaffolded *de novo* genome assembly was evaluated using BUSCO genes (embryophyta_odb10; 2020-09-10; Supplementary File S4) and resulted in a completeness score of 87.7%. Other large genome assemblies, such as the *loblolly pine* v1.01 genome has an CEGMA completeness of 91% (Neale *et al.* 2014) and the *Allium sativum* v1.0 genome has an CEGMA completeness score of 92.7% and a BUSCO completeness score of 88.7% (Sun *et al.* 2020). Of the BUSCO genes, 3.3% were labeled as fragmented, which could be because the length of the gene model did not fall within the expected length distribution of the chosen BUSCO profile. Also technical limitation of the algorithm might increase proportions of fragmented and missing BUSCOs, especially in large genomes (Simao *et al.* 2015). Still, the slightly lower completeness scores in onion suggests that we miss genes in our assembly.

Initial repeat masking of the genome with repeatmasker, using the REPBASE v20.5 database, resulted in 15.1% of the genome to be annotated as repetitive (Supplementary Table S5), which is far below the expected 95% moderate to high repetitive regions (Flavell *et al.* 1974) though comparable to homology-based repeat annotation in other large genomes, such as *loblolly pine* (Wegrzyn *et al.* 2013). Though we expect REPBASE to be missing *Allium* specific repeats, this result indicates that most repeats in the genome are not recognizable anymore as repeats, have accumulated mutations and/or degraded due to repeated transpositions within a transposon and therefore disturbing its structure, and are probably (very) old. This observation agrees with the observed Kmer statistics, which suggests that 53.8% of the genome is single copy and the observations by Jakše *et al.* (2008) that onion BAC sequences contain >50% sequences that are like transposons, many of which are degraded. Vitte *et al.* (2013) showed that current repeat databases contain limited data from onion-related species, hampering homology-based detection of TE’s, which is supported by our finding that <10% of the long terminal repeat (LTR) retrotransposons could be directly identified in such an approach. Using *de novo* repeat annotation strategies, genomes comparable in size, such as garlic (Sun *et al.* 2020), bread wheat (Mayer *et al.* 2014), and loblolly pine (Wegrzyn *et al.* 2014) were annotated as containing approx. 91%, 80%, and 82% repetitive DNA sequences, respectively. Therefore, we used the “all vs all comparison methods,” as implemented in the REPET pipeline (Flutre *et al.* 2011) to develop an onion-specific repeat database. Using this *de novo* developed repeat database, 72.4% of the genome sequence could be classified as repetitive and was subsequently masked for downstream analysis. Still, this percentage is lower than the expected 95%, suggesting that the remaining ∼20% of the “repetitive” sequences were too diverged to be recognized. This touches upon a standing debate within the Onion community on the age of onion-LTR’s. Jakše *et al.* (2008) suggests that most LTRs are old and inactive while Vitte *et al.* (2013) suggests that young, intact and nested LTRs are abundant in the onion genome. Our results indicate that nesting of elements of LTR sequences occurs frequently, in line with the study of Vitte *et al* (2013). Our genome assembly can serve as a template to further investigate this question.

LTR retrotransposons are the major contributors to the size of the onion genome (Supplementary Table S5), which is in line with previous studies (Jakše *et al.* 2008; Vitte *et al.* 2013). The REPBASE annotation indicates that the majority of the young LTRs are of the Gypsy type; followed by Copia. This has also been observed in other large plant genomes such as spruce (Nystedt *et al.* 2013), garlic (Sun *et al.* 2020), and wheat (Appels *et al.* 2018). LTR retrotransposons have been described in relation to genome size increase (Kumar and Bennetzen 1999) and have been suggested to play a significant role in adaptive response of the genome to environmental challenges (McClintock 1984).

*Ab initio* prediction of gene models on the repeat masked genome sequence with Augustus resulted in the identification of 540,925 gene models, from which 47,066 showed >90% coverage with reads from the RNAseq dataset (Table 1). The number of gene models is way beyond the average number of genes of 36,795 reported for plant genomes (Ramírez-Sánchez *et al.* 2016). A proper annotation to identify only functional genes would require extensive manual curation (Hosmani *et al.* 2019b; Tello-Ruiz *et al.* 2019; Athanasouli *et al.* 2020). We decided to make the extended set available for the community, rather than restricting ourselves to making only models available with additional RNAseq support. The abundance of gene models may most likely be explained by the presence of pseudogenes (Zhang and Gerstein 2004; Xiao *et al.* 2016). Pseudogenes are non-functional copies of genes that were once active in the ancestral genome. For example, in Arabidopsis 924 pseudogenes are known (Xiao *et al.* 2016) while in wheat 288,839 pseudogenes were identified (Appels *et al.* 2018). A pseudogene still has characteristics of a gene and will be detected using an *ab initio* gene model prediction algorithm, but not be annotated by blast against curated protein databases, such as TrEMBL, due to partial matches. Blast analysis against TrEMBL resulted in hits for 86,073 gene models (15.9% of the *ab initio* predicted models). Using Blast2Go (Conesa *et al.* 2005), 88,259 gene models were functionally annotated, of which 49,918 models were annotated using data from both Blast and InterPro. Of these, 17,457 models were annotated exclusively by InterPro, while 20,884 models had a blast hit only. For subsequent analysis, we focus on the subset of 86,073 models with similarity to genes from TrEMBL, of which 25,344 showed >90% coverage with reads from the RNAseq dataset. The average coding sequence length of onion genes is 879 bp and is spread over 4.4 exons. This is shorter than the average plant gene length of 1308 bp (Ramírez-Sánchez *et al.* 2016), though still within the observed variation and larger than the average gene length of 797 bp reported for garlic (Sun *et al.* 2020). Average intron length is 1035 bp (Table 1), though the largest predicted intron is 213 Kb. Although a positive relationship between intron size and genome size has been observed (Stival Sena *et al.* 2014), due to large variations that occur in intron sizes, it is not a good predictor for genome size (Wendel *et al.* 2002). With a median and average intron size of 178 and 1035 bp, respectively, the majority of introns in onion is short, while a limited number of introns is (very) long. This is probably because of the energy required for transcribing the long genes.

### Organization of the onion gene space

**Figure 1:**
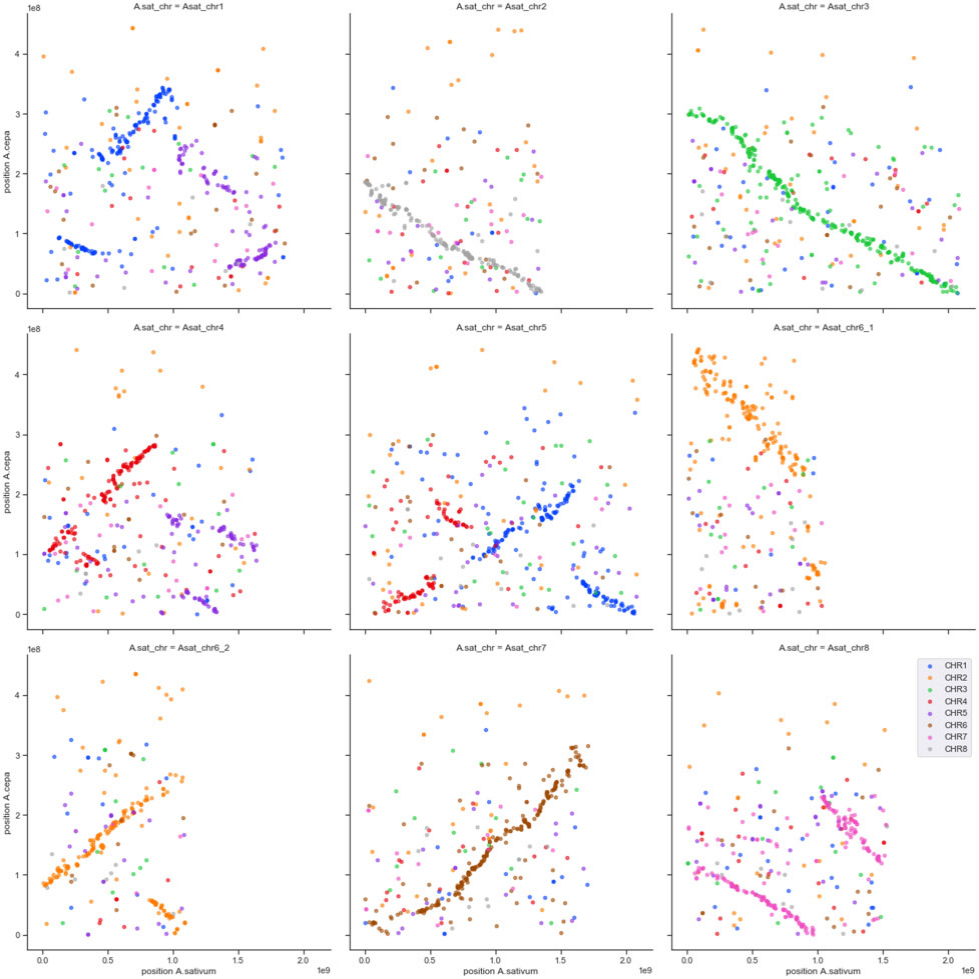
Physical positions of the marker on the garlic pseudomolecules (*x*-axis) plotted against the physical position of the marker on the onion pseudomolecules (*y*-axis). Color of the points represent the original linkage group assignment of the marker on the onion genetic map. Synteny between the garlic and onion genome can be observed in several chromosomes (*e.g.*, garlic chromosome 2, 3, and 7 and its onion counterparts chromosome 8, 3, chr6, respectively) though signals of translocation can also be observed (*e.g.*, garlic chr5 and parts of onion chromosome 1 and 4). The data suggest inversions within a chromosome (*e.g.*, garlic chromosome 7 and its onion counterpart chromosome 6), but this cannot be estimated with certainty as not all our contigs could be oriented using Allmaps.

In plants, we have seen two scenario’s for the distribution of genes: genes mainly located in actively recombining euchromatin regions, while large non-recombining regions (centromeres) have a low gene content, such as in tomato (Hosmani *et al.* 2019a), or genes equally distributed over the genome, such as in garlic (Sun *et al.* 2020). If onion would show a similar pattern as tomato, then most gene models will be included in the current set of pseudomolecules. Based on the set of 86 K models with a match to the TrEMBL database, we calculated a rate of 8108 and 5352 gene models/Gb for anchored and unanchored scaffolds, respectively. This shows that gene density in the anchored scaffolds is approximately 1.5× higher than in the unanchored scaffolds. In tomato, we calculated gene density in euchromatin and heterochromatin (Sim *et al.* 2012; Víquez-Zamora *et al.* 2014) to be 97,715 and 20,010 gene models/Gb respectively, a difference of approximately 4.9×. Interpretation of this data and the meaning for onion must be treated with caution as it is influenced by two factors; (1) the difference in genome size between onion (16 Gb) and tomato (850 Mb) and (2) the fact that only 2.4 Gb (out of 14.9 Gb) of the onion scaffolds are yet organized into pseudomolecules. Having said that, like Jakše et al. (2008) who previously sequenced two onion BACs, we hypothesize that genes in onion are more equally distributed over the genome, and reside in an ocean of repetitive elements, as the ratio between scaffolds incorporated in pseudomolecules and the scaffolds not incorporated in pseudomolecules is much closer to 1, than the ratio observed in tomato. This hypothesis is further supported by the results of the synteny analysis with garlic. Not only the 3515 of the 5339 onion unigene derived EST markers (Fujito *et al.* 2021) showed a uniform distribution on the garlic and onion pseudomolecules (Figure 1), also the distribution of transposable elements is uniform over the garlic and onion chromosomes (Sun *et al.* 2020; Supplementary Figure S7). Our hypothesis is in line with previous results in which the mapping of genes on physical chromosomes using molecular cytogenetic methods showed that the genes in *Allium* are localized in all three regions of the chromosome arms: proximal, interstitial, and distal (Scholten *et al.* 2007; Masamura *et al.* 2012; Khrustaleva *et al.* 2016, 2019a,b).

### Synteny between *Allium cepa* (onion) and *Allium sativum* (garlic)

The sequence of 5339 Onion unigenes (Fujito *et al.* 2021) were also used to study the synteny between onion and garlic (Sun *et al.* 2020). For 3515 unigenes, physical positions were determined on both the garlic and onion pseudomolecules. Overall synteny between some chromosomes is strong, such as for garlic chromosomes 2, 3, and 7 and their onion counterparts’ chromosome 8, 3, and 6, respectively (Figure 1). Signals for translocations between chromosomes were also observed, as garlic chromosome 5 seems to be split over onion chromosome 1 and 4. In addition, signals for inversions were observed, for instance for garlic chromosome 7 (onion chromosome 6). Where the signal for syntenic relationships between garlic and onion genomes are high, the garlic genome sequence may be used to further organize the onion scaffolds into pseudomolecules.

### Improving usability of the onion genome assembly

The onion genome assembly in its current form is already a powerful tool for research and practical breeding. The annotation will be a good starting point for mining the genome for candidate genes while the set of pseudomolecules will facilitate the development of new markers for targeted regions. As syntenic relationships between garlic and onion genomes are high, insight in the overall synteny between garlic and onion can be used to develop hypothesis and assign unplaced scaffolds to approximate positions on the onion pseudomolecules, further facilitating discovery of novel insights. However, real improvements should come from additional lab work. Our current assembly is primarily based on Illumina short read sequencing and covers approx. 91% of the expected genome size and has a BUSCO completeness value 87.7% indicating, this assembly still needs improvement. Current third- generation sequencing technologies, such as ONT long read (Jain *et al.* 2016) and PacBio HiFi (Wenger *et al.* 2019), have shown to deliver larger continues genome assemblies (Michael and VanBuren 2020). Data from one or both platforms, combined with, for example, Hi-C scaffolding data, would lead to a more continuous assembly, as shown for garlic (Sun *et al.* 2020), a genome with a size similar to onion.

### Perspective for addressing evolutionary questions regarding genome size expansion

With the development of genome assemblies in the *Alliaceae*, but also in other groups of plant species with large genomes, for example, gymnosperms (Zimin *et al.* 2014), data are becoming available to study genome size expansion. Earlier studies in onion, suggests that tandem duplications plays a role (King *et al.* 1998). The garlic genome assembly shows the presence of intra and inter chromosome syntenic blocks (Sun *et al.* 2020). The onion pseudogenes will likely contain signals supporting either tandem and/or segmental duplication. We have developed a partly annotated genome-specific repeat database, with the purpose to mask the genome for *ab initio* gene prediction. The study of Vitte *et al.* (2013) has already shown that homology-based annotation using reference libraries will likely miss intact LTRs, such as onion-specific GYPSY and COPIA, and that further annotation is needed using the know domain structure of retroelements (Wicker *et al.* 2007). Such analysis will give further insights in the history of repeats involved in genome expansion. The comparison of (pseudo)gene and ancient retroelements to, for example, Garlic and Loblolly pine will be a next step in understanding genome size expansion in onion, but also in other plant species with large genomes.

## Conclusion

We have produced the first *de novo* genome sequence of onion. The sequence provides insights into the distribution of genes and repeats in this important cop species. This assembly is the first high-quality genome sequence and will be a valuable resource for both research and breeding.

## References

[jkab243-B1] Abdelrahman M , El-Sayed M, Sato S, Hirakawa H, Ichi Ito S, et al 2017. RNA-sequencing-based transcriptome and biochemical analyses of steroidal saponin pathway in a complete set of *Allium fistulosum*—*A. cepa* monosomic addition lines. PLoS One. 12:e0181784. 28800607 10.1371/journal.pone.0181784 PMC5553718

[jkab243-B2] Appels R , Eversole K, Feuillet C, Keller B, Rogers J, et al 2018. Shifting the limits in wheat research and breeding using a fully annotated reference genome. Science. 361:eaar7191. 30115783 10.1126/science.aar7191

[jkab243-B3] Arumuganathan K , Earle ED. 1991. Nuclear DNA content of some important plant species. Plant Mol Biol Rep. 9:208–218.

[jkab243-B4] Athanasouli M , Witte H, Weiler C, Loschko T, Eberhardt G, et al 2020. Comparative genomics and community curation further improve gene annotations in the nematode Pristionchus pacificus. BMC Genomics. 21:1–9. 10.1186/s12864-020-07100-0 PMC7552371 33045985

[jkab243-B5] Baldwin S , Pither-Joyce M, Wright K, Chen L, McCallum J. 2012. Development of robust genomic simple sequence repeat markers for estimation of genetic diversity within and among bulb onion (*Allium cepa* L.) populations. Mol Breeding. 30:1401–1411.

[jkab243-B5633014] Bernatzky R , Tanksley SD. 1986. Toward a saturated linkage map in tomato based on isozymes and random cDNA sequences. Genetics. 112:887–898. 17246322 10.1093/genetics/112.4.887 PMC1202783

[jkab243-B6] Brenchley R , Spannagl M, Pfeifer M, Barker GLA, D'Amore R, et al 2012. Analysis of the bread wheat genome using whole-genome shotgun sequencing. Nature. 491:705–710. 23192148 10.1038/nature11650 PMC3510651

[jkab243-B7] Choi Y , Kim S, Lee J. 2020. Construction of an onion (*Allium cepa* L.) genetic linkage map using genotyping-by-sequencing analysis with a reference gene set and identification of qtls controlling anthocyanin synthesis and content. Plants. 9:616–617. 10.3390/plants9050616 PMC7285762 32408580

[jkab243-B8] Conesa A , Götz S, García-Gómez JM, Terol J, Talón M, et al 2005. Blast2GO: A universal tool for annotation, visualization and analysis in functional genomics research. Bioinformatics. 21:3674–3676. 16081474 10.1093/bioinformatics/bti610

[jkab243-B9] Dobin A , Davis CA, Schlesinger F, Drenkow J, Zaleski C, et al 2013. STAR: Ultrafast universal RNA-seq aligner. Bioinformatics. 29:15–21. 23104886 10.1093/bioinformatics/bts635 PMC3530905

[jkab243-B10] Duangjit J , Bohanec B, Chan AP, Town CD, Havey MJ. 2013. Transcriptome sequencing to produce SNP-based genetic maps of onion. Theor Appl Genet. 126:2093–2101. 23689743 10.1007/s00122-013-2121-x

[jkab243-B11] Flavell RB , Bennett MD, Smith JB, Smith DB. 1974. Genome size and the proportion of repeated nucleotide sequence DNA in plants. Biochem Genet. 12:257–269. 4441361 10.1007/BF00485947

[jkab243-B12] Flutre T , Duprat E, Feuillet C, Quesneville H. 2011. Considering transposable element diversification in *de novo* annotation approaches (Y. Xu, Ed.). PLoS One. 6:e16526. 21304975 10.1371/journal.pone.0016526 PMC3031573

[jkab243-B13] Fujito S , Akyol TY, Mukae T, Wako T, Yamashita K, et al 2021. Construction of a high-density linkage map and graphical representation of the arrangement of transcriptome-based unigene markers on the chromosomes of onion, *Allium cepa* L. BMC Genomics. 22:481. 34174821 10.1186/s12864-021-07803-y PMC8236188

[jkab243-B14] Gotz S , Garcia-Gomez JM, Terol J, Williams TD, Nagaraj SH, et al 2008. High-throughput functional annotation and data mining with the Blast2GO suite. Nucleic Acids Res. 36:3420–3435. 18445632 10.1093/nar/gkn176 PMC2425479

[jkab243-B15] Harkess A , Zhou J, Xu C, Bowers JE, Van der Hulst R, et al 2017. The asparagus genome sheds light on the origin and evolution of a young Y chromosome. Nat. Commun. 8:1279. 29093472 10.1038/s41467-017-01064-8 PMC5665984

[jkab243-B16] Havey MJ , McCallum J. 2012. An international plan for sequencing and annotation of onion. https://haveylab.horticulture.wisc.edu/wp-content/uploads/sites/66/2016/07/Onion-Sequencing-White-Paper-Final.pdf.

[jkab243-B17] Hoff KJ , Lange S, Lomsadze A, Borodovsky M, Stanke M. 2015. BRAKER1: unsupervised RNA-seq-based genome annotation with GeneMark-ET and AUGUSTUS: Table 1. Bioinformatics. 32:767–769. 26559507 10.1093/bioinformatics/btv661 PMC6078167

[jkab243-B18] Hosmani PS , Flores-Gonzalez M, van de Geest H, Maumus F, Bakker LV, et al 2019a. An improved de novo assembly and annotation of the tomato reference genome using single-molecule sequencing, Hi-C proximity ligation and optical maps. bioRxiv 2012: 767764.

[jkab243-B19] Hosmani PS , Shippy T, Miller S, Benoit JB, Munoz-Torres M, et al 2019b. A quick guide for student-driven community genome annotation. PLoS Comput Biol. 15:1–11. 10.1371/journal.pcbi.1006682 PMC6447164 30943207

[jkab243-B20] Hyde P , Earle E, Mutschler M. 2012. Doubled haploid onion (*Allium cepa* L.) lines and their impact on hybrid performance. HortScience. 47:1690–1695.

[jkab243-B21] Jain M , Olsen HE, Paten B, Akeson M. 2016. The Oxford Nanopore MinION: delivery of nanopore sequencing to the genomics community. Genome Biol. 17:239. 27887629 10.1186/s13059-016-1103-0 PMC5124260

[jkab243-B22] Jakše J , Meyer JDF, Suzuki G, McCallum J, Cheung F, et al 2008. Pilot sequencing of onion genomic DNA reveals fragments of transposable elements, low gene densities, and significant gene enrichment after methyl filtration. Mol Genet Genomics. 280:287–292. 18615255 10.1007/s00438-008-0364-z

[jkab243-B23] Jones P , Binns D, Chang H-Y, Fraser M, Li W, et al 2014. InterProScan 5: genome-scale protein function classification. Bioinformatics. 30:1236–1240. 24451626 10.1093/bioinformatics/btu031 PMC3998142

[jkab243-B24] Kamenetsky R , Faigenboim A, Shemesh Mayer E, Michael TB, Gershberg C, et al 2015. Integrated transcriptome catalogue and organ-specific profiling of gene expression in fertile garlic (*Allium sativum* L.). BMC Genomics. 16:12. 25609311 10.1186/s12864-015-1212-2 PMC4307630

[jkab243-B25] Kelly LJ , Leitch IJ. 2011. Exploring giant plant genomes with next-generation sequencing technology. Chromosome Res. 19:939–953. 21987187 10.1007/s10577-011-9246-z

[jkab243-B26] Khrustaleva L , Jiang J, Havey MJ. 2016. High-resolution tyramide-FISH mapping of markers tightly linked to the male-fertility restoration (Ms) locus of onion. Theor Appl Genet. 129:535–545. 26704420 10.1007/s00122-015-2646-2

[jkab243-B27] Khrustaleva L , Kudryavtseva N, Romanov D, Ermolaev A, Kirov I. 2019a. Comparative Tyramide-FISH mapping of the genes controlling flavor and bulb color in *Allium* species revealed an altered gene order. Sci Rep. 9:12007. 31427665 10.1038/s41598-019-48564-9 PMC6700127

[jkab243-B28] Khrustaleva L , Mardini M, Kudryavtseva N, Alizhanova R, Romanov D, et al 2019b. The power of genomic in situ hybridization (GISH) in interspecific breeding of bulb onion (*Allium cepa* L.) resistant to Downy Mildew (Peronospora destructor [Berk.] Casp.). Plants. 8:36. 10.3390/plants8020036 PMC6410304 30720753

[jkab243-B29] Kim S , Kim M-S, Kim Y-M, Yeom S-I, Cheong K, et al 2014. Integrative structural annotation of de novo RNA-Seq provides an accurate reference gene set of the enormous genome of the onion (*Allium cepa* L.). DNA Res. 22:19–27. 25362073 10.1093/dnares/dsu035 PMC4379974

[jkab243-B30] King JJ , Bradeen JM, Bark O, McCallum JA, Havey MJ. 1998. A low-density genetic map of onion reveals a role for tandem duplication in the evolution of an extremely large diploid genome. Theor Appl Genet. 96:52–62.

[jkab243-B31] Kuhl JC , Havey MJ, Martin WJ, Cheung F, Yuan Q, et al 2005. Comparative genomic analyses in Asparagus. Genome. 48:1052–1060. 16391674 10.1139/g05-073

[jkab243-B32] Kumar A , Bennetzen JL. 1999. Plant retrotransposons. Annu Rev Genet. 33:479–532. 10690416 10.1146/annurev.genet.33.1.479

[jkab243-B33] Kumar A , Pearce SR, McLean K, Harrison G, Heslop-Harrison JS, et al 1997. The Ty1-copia group of retrotransposons in plants: genomic organisation, evolution, and use as molecular markers. Genetica. 100:205–217. 9440274

[jkab243-B34] Marçais G , Kingsford C. 2011. A fast, lock-free approach for efficient parallel counting of occurrences of k-mers. Bioinformatics. 27:764–770. 21217122 10.1093/bioinformatics/btr011 PMC3051319

[jkab243-B35] Marcussen T , Sandve SR, Heier L, Spannagl M, Pfeifer M, et al, International Wheat Genome Sequencing Consortium. 2014. Ancient hybridizations among the ancestral genomes of bread wheat. Science. 345:1250092. 25035499 10.1126/science.1250092

[jkab243-B36] Martin WJ , McCallum J, Shigyo M, Jakse J, Kuhl JC, et al 2005. Genetic mapping of expressed sequences in onion and in silico comparisons with rice show scant colinearity. Mol Genet Genomics. 274:197–204. 16025250 10.1007/s00438-005-0007-6

[jkab243-B37] Masamura N , McCallum J, Khrustaleva L, Kenel F, Pither-Joyce M, et al 2012. Chromosomal organization and sequence diversity of genes encoding lachrymatory factor synthase in *Allium cepa* L. G3 (Bethesda). 2:643–651. 22690373 10.1534/g3.112.002592 PMC3362293

[jkab243-B38] Mayer KFX , Rogers J, Dole el J, Pozniak C, Eversole K , et al; The International Wheat Genome Sequencing Consortium (IWGSC). 2014. A chromosome-based draft sequence of the hexaploid bread wheat (*Triticum aestivum*) genome. Science. 345:1251788. 25035500 10.1126/science.1251788

[jkab243-B39] McCallum J , Baldwin S, Shigyo M, Deng Y, van Heusden S, et al 2012. AlliumMap-A comparative genomics resource for cultivated *Allium* vegetables. BMC Genomics. 13:168. 22559261 10.1186/1471-2164-13-168 PMC3423043

[jkab243-B40] McClintock B. 1984. The significance of responses of the genome to challenge. Science. 226:792–801. 15739260 10.1126/science.15739260

[jkab243-B41] Michael TP , VanBuren R. 2020. Building near-complete plant genomes. Curr Opin Plant Biol. 54:26–33. 31981929 10.1016/j.pbi.2019.12.009

[jkab243-B42] Neale DB , Wegrzyn JL, Stevens K. A, Zimin AV, Puiu D, et al 2014. Decoding the massive genome of loblolly pine using haploid DNA and novel assembly strategies. Genome Biol. 15:R59. 24647006 10.1186/gb-2014-15-3-r59 PMC4053751

[jkab243-B43] Nystedt B , Street NR, Wetterbom A, Zuccolo A, Lin Y-C, et al 2013. The Norway spruce genome sequence and conifer genome evolution. Nature. 497:579–584. 23698360 10.1038/nature12211

[jkab243-B44] Pearce SR , Pich U, Harrison G, Flavell AJ, Heslop-Harrison JS, et al 1996. The Ty1-copia group retrotransposons of *Allium cepa* are distributed throughout the chromosomes but are enriched in the terminal heterochromatin. Chromosome Res. 4:357–364. 8871824 10.1007/BF02257271

[jkab243-B45] Pich U , Schubert I. 1998. Terminal heterochromatin and alternative telomeric sequences in *Allium cepa*. Chromosom. Res. 6:315–321. 10.1023/a:1009227009121 9688522

[jkab243-B46] Putnam NH , O'Connell BL, Stites JC, Rice BJ, Blanchette M, et al 2016. Chromosome-scale shotgun assembly using an in vitro method for long-range linkage. Genome Res. 26:342–350. 26848124 10.1101/gr.193474.115 PMC4772016

[jkab243-B47] Quesneville H , Bergman CM, Andrieu O, Autard D, Nouaud D, et al 2005. Combined evidence annotation of transposable elements in genome sequences. PLoS Comp Biol. 1:e22. 10.1371/journal.pcbi.0010022 PMC1185648 16110336

[jkab243-B48] Ramírez-Sánchez O , Pérez-Rodríguez P, Delaye L, Tiessen A. 2016. Plant proteins are smaller because they are encoded by fewer exons than animal proteins. Genomics Proteomics Bioinformatics. 14:357–370. 27998811 10.1016/j.gpb.2016.06.003 PMC5200936

[jkab243-B49] Ricroch A , Yockteng R, Brown SC, Nadot S. 2005. Evolution of genome size across some cultivated *Allium* species. Genome. 48:511–520. 16121247 10.1139/g05-017

[jkab243-B50] Scholten OE , van Heusden AW, Khrustaleva LI, Burger-Meijer K, Mank RA, et al 2007. The long and winding road leading to the successful introgression of downy mildew resistance into onion. Euphytica. 156:345–353.

[jkab243-B51] Scholten OE , van Kaauwen MPW, Shahin A, Hendrickx PM, Keizer LCP, et al 2016. SNP-markers in *Allium* species to facilitate introgression breeding in onion. BMC Plant Biol. 16:187. 27576474 10.1186/s12870-016-0879-0 PMC5006257

[jkab243-B52] Shibata F , Hizume M. 2002. The identification and analysis of the sequences that allow the detection of *Allium cepa* chromosomes by GISH in the allodiploid A. wakegi. Chromosoma. 111:184–191. 12355208 10.1007/s00412-002-0197-1

[jkab243-B53] Shigyo M , Tashiro Y, Isshiki S, Miyazaki S. 1996. Establishment of a series of alien monosomic addition lines of Japanese bunching onion (*Allium fistulosum* L.) with extra chromosomes from shallot (*A. cepa L. aggregatum* group). Genes Genet Syst. 71:363–371. 9080683 10.1266/ggs.71.363

[jkab243-B54] Sim S-C , Durstewitz G, Plieske J, Wieseke R, Ganal MW, et al 2012. Development of a large SNP genotyping array and generation of high-density genetic maps in tomato (T. Yin, Ed.). PLoS One. 7:e40563. 22802968 10.1371/journal.pone.0040563 PMC3393668

[jkab243-B55] Simao FA , Waterhouse RM, Ioannidis P, Kriventseva EV, Zdobnov EM. 2015. BUSCO: assessing genome assembly and annotation completeness with single-copy orthologs. Bioinformatics. 31:3210–3212. 26059717 10.1093/bioinformatics/btv351

[jkab243-B56] Skinner ME , Uzilov AV, Stein LD, Mungall CJ, Holmes IH. 2009. JBrowse: a next-generation genome browser. Genome Res. 19:1630–1638. 19570905 10.1101/gr.094607.109 PMC2752129

[jkab243-B57] Sohn S-H , Ahn Y-K, Lee T-H, Lee J-E, Jeong M-H, et al 2016. Construction of a draft reference transcripts of onion (*Allium cepa*) using long-read sequencing. Plant Biotechnol Rep. 10:383–390.

[jkab243-B58] Stack SM , Comings DE. 1979. The chromosomes and DNA of *Allium cepa*. Chromosoma. 70:161–181.

[jkab243-B59] Stanke M , Diekhans M, Baertsch R, Haussler D. 2008. Using native and syntenically mapped cDNA alignments to improve *de novo* gene finding. Bioinformatics. 24:637–644. 18218656 10.1093/bioinformatics/btn013

[jkab243-B60] Stival Sena J , Giguère I, Boyle B, Rigault P, Birol I, et al 2014. Evolution of gene structure in the conifer Picea glauca: a comparative analysis of the impact of intron size. BMC Plant Biol. 14:95. 24734980 10.1186/1471-2229-14-95 PMC4108047

[jkab243-B61] Sun X , Zhu S, Li N, Cheng Y, Zhao J, et al 2020. A chromosome-level genome assembly of garlic (*Allium sativum*) provides insights into genome evolution and allicin biosynthesis. Mol Plant. 13:1328–1339. 32730994 10.1016/j.molp.2020.07.019

[jkab243-B62] Tang H , Zhang X, Miao C, Zhang J, Ming R, et al 2015. ALLMAPS: robust scaffold ordering based on multiple maps. Genome Biol. 16:3. 25583564 10.1186/s13059-014-0573-1 PMC4305236

[jkab243-B63] Tello-Ruiz MK , Marco CF, Hsu FM, Khangura RS, Qiao P, et al 2019. Double triage to identify poorly annotated genes in maize: the missing link in community curation. bioRxiv. 1–13. 10.1371/journal.pone.0224086 PMC6816542 31658277

[jkab243-B64] *Van Heusden AW, Shigyo M, Tashiro Y, Vrielink-Van Ginkel R, Kik C. 2000 AFLP linkage group assignment to the chromosomes of *Allium cepa* L. via monosomic addition lines. Theor Appl Genet. 100:480–486.

[jkab243-B65] Van’T Hof J. 1965. Relationships between mitotic cycle duration, S period duration and the average rate of DNA synthesis in the root meristem cells of several plants. Exp Cell Res. 39:48–58. 5831250 10.1016/0014-4827(65)90006-6

[jkab243-B66] Víquez-Zamora M , Caro M, Finkers R, Tikunov Y, Bovy A, et al 2014. Mapping in the era of sequencing: high density genotyping and its application for mapping TYLCV resistance in *Solanum pimpinellifolium*. BMC Genomics. 15:1152. 25526885 10.1186/1471-2164-15-1152 PMC4367842

[jkab243-B67] Vitte C , Estep MC, Leebens-Mack J, Bennetzen JL. 2013. Young, intact and nested retrotransposons are abundant in the onion and asparagus genomes. Ann Bot. 112:881–889. 23887091 10.1093/aob/mct155 PMC3747808

[jkab243-B68] von Kohn C , Kiełkowska A, Havey MJ. 2013. Sequencing and annotation of the chloroplast DNAs and identification of polymorphisms distinguishing normal male-fertile and male-sterile cytoplasms of onion. Genome. 56:737–742. 24433209 10.1139/gen-2013-0182

[jkab243-B69] Wegrzyn JL , Liechty JD, Stevens K. A, Wu L-S, Loopstra C. A, et al 2014. Unique features of the loblolly pine (*Pinus taeda* L.) megagenome revealed through sequence annotation. Genetics. 196:891–909. 24653211 10.1534/genetics.113.159996 PMC3948814

[jkab243-B70] Wegrzyn JL , Lin BY, Zieve JJ, Dougherty WM, Martínez-García PJ, et al 2013. Insights into the loblolly pine genome: characterization of BAC and fosmid sequences. PLoS One. 8:e72439. 24023741 10.1371/journal.pone.0072439 PMC3762812

[jkab243-B71] Wendel JF , Cronn RC, Alvarez I, Liu B, Small RL, et al 2002. Intron size and genome size in plants. Mol Biol Evol. 19:2346–2352. 12446829 10.1093/oxfordjournals.molbev.a004062

[jkab243-B72] Wenger AM , Peluso P, Rowell WJ, Chang PC, Hall RJ, et al 2019. Accurate circular consensus long-read sequencing improves variant detection and assembly of a human genome. Nat Biotechnol. 37:1155–1162. 31406327 10.1038/s41587-019-0217-9 PMC6776680

[jkab243-B73] Wicker T , Sabot F, Hua-Van A, Bennetzen JL, Capy P, et al 2007. A unified classification system for eukaryotic transposable elements. Nat Rev Genet. 8:973–982. 17984973 10.1038/nrg2165

[jkab243-B74] Xiao J , Kumarsekhwal M, Li P, Raja R, Sylvie C, et al 2016. Pseudogenes and their genome-wide prediction in plants. Int J Mol Sci. 17:1991. 10.3390/ijms17121991 PMC5187791 27916797

[jkab243-B75] Ye C , Hill CM, Wu S, Ruan J, Ma Z. 2016. DBG2OLC: efficient assembly of large genomes using long erroneous reads of the third generation sequencing technologies. Sci Rep. 6:31900. 27573208 10.1038/srep31900 PMC5004134

[jkab243-B76] Zhang Z , Gerstein M. 2004. Large-scale analysis of pseudogenes in the human genome. Curr Opin Genet Dev. 14:328–335. 15261647 10.1016/j.gde.2004.06.003

[jkab243-B77] Zimin A , Stevens K. A, Crepeau MW, Holtz-Morris A, Koriabine M, et al 2014. Sequencing and assembly of the 22-Gb loblolly pine genome. Genetics. 196:875–890. 24653210 10.1534/genetics.113.159715 PMC3948813

[jkab243-B78] Zimin AV , Marçais G, Puiu D, Roberts M, Salzberg SL, et al 2013. The MaSuRCA genome assembler. Bioinformatics. 29:2669–2677. 23990416 10.1093/bioinformatics/btt476 PMC3799473

